# Rat-Bite Fever in Human with *Streptobacillus notomytis* Infection, Japan

**DOI:** 10.3201/eid2407.171580

**Published:** 2018-07

**Authors:** Yoshihiko Ogawa, Kei Kasahara, Sang-Tae Lee, Takamitsu Ito, Hideo Hasegawa, Sachie Hirose, Shigeru Santo, Atsushi Yoshida, Ryuichi Nakano, Hisakazu Yano, Keiichi Mikasa

**Affiliations:** Nara Medical University, Nara, Japan (Y. Ogawa, K. Kasahara, S.T. Lee, R. Nakano, H. Yano, K. Mikasa);; Osaka Gyoumeikan Hospital, Osaka, Japan (T. Ito, H. Hasegawa, S. Hirose, S. Santo, A. Yoshida)

**Keywords:** rat-bite fever, *Streptobacillus notomytis*, *Streptobacillus moniliformis*, 16S ribosomal RNA gene, bacteria, zoonoses, Japan, streptococci

## Abstract

We report a case of rat-bite fever in a 94-year-old woman with *Streptobacillus notomytis* infection. We established an epidemiologic link between exposure to rats and human infection by performing nested PCRs that detected *S. notomytis* in the intraoral swab specimens obtained from rats captured in the patient’s house.

*Streptobacillus* is a genus of gram-negative, filamentous, rod-shaped bacilli belonging to the family *Leptotrichiaceae*. Since 2014, four novel species other than *S. moniliformis* have been reported: *S. hongkongensis* was isolated from 2 human patients, *S. felis* from the lung of a cat, *S. ratti* from black rats, and *S. notomytis* from a spinifex hopping mouse (*1*–*4*). We report a case of a human infection with *S. notomytis*.

A 94-year-old woman sought treatment at our hospital for general malaise, anorexia, and bilateral knee pain. At admission, her body temperature was 38°C; physical examination revealed swelling in both knees. Her skin was intact, with no rashes or animal bites. Laboratory tests revealed high leukocyte count (1.42 × 10^9^ cells/L) and elevated level of C-reactive protein (19.5 mg/dL).

Bilateral knee arthrocentesis yielded 25 mL of purulent fluid; Gram stain demonstrated the presence of few, thin, gram-negative bacilli with pyrophosphate calcium crystals and neutrophils (Figure). Bacterial culture yielded transparent, small, smooth colonies on 5% sheep blood agar (Kyokuto, Tokyo, Japan) incubated at 37°C under 5% CO_2_ for 48 h. However, the automated bacterial identification method (Vitek 2; bioMérieux, Tokyo, Japan) failed to identify the isolate. We evaluated the isolate (NR2245) by matrix-assisted laser desorption/ionization time-of-flight mass spectrometry using Bruker MALDI BioTyper software version 4.001 library database (Bruker Daltonik GmbH, Bremen, Germany) employing ethanol–formic acid extraction. We identified the isolate as *S. moniliformis* (score: 1.608, 24 h). The database included only 1 entry from *S. moniliformis*, DSM 12112T.

**Figure:**
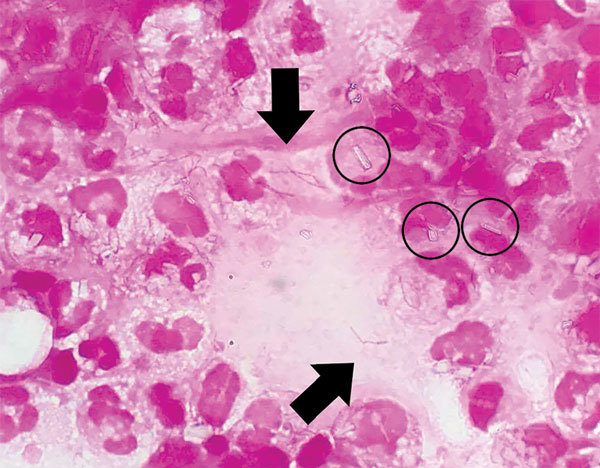
Gram staining of pus obtained from a patient with rat-bite fever. Circles indicate pyrophosphate calcium crystals. Arrows indicate chain-shaped gram-negative bacilli. Original magnification ×1,000.

We administered ceftriaxone. Subsequent results of arthrocentesis and blood cultures (BacT/ALERT; bioMérieux) were negative; however, the patient’s fever and bilateral knee pain persisted. Transthoracic echocardiography showed no evidence of infective endocarditis. We replaced ceftriaxone with sulbactam and ampicillin on hospital day 16, followed by intraarticular administration of dexamethasone on day 17 for pseudogout (diagnosed by the presence of pyrophosphate calcium crystals). On day 20, we performed bilateral knee lavage; thereafter, the patient’s fever and knee pain resolved. The surgery specimen was serous fluid; results of Gram stain and aerobic and anaerobic culture were negative. On day 30, we replaced sulbactam/ampicillin treatment with oral minocycline (100 mg every 12 h) as maintenance therapy; however, pneumonia developed, and the patient died of respiratory failure on day 56. We detected *Acinetobacter baumannii* complex and *Enterococcus faecium* from the sputum; however, we did not detect *Streptobacillus* species.

To identify the isolate from the patient’s synovial fluid, we performed 16S rRNA gene sequencing using a universal primer pair: 27F (5′-AGAGTTTGATCC TGGCTCAG-3′) and 1492R (5′-GGTTACCTTGTTACGACTT-3′). The sequence (GenBank accession no. LC360808) showed 100% identity (1,380/1,380 bp) to *S. notomytis* AHL_370–1^T^ (GenBank accession no. KR001919) and 98.55% (1,360/1,380 bp) identity to *S. moniliformis* DSM12112^T^ (GenBank accession no. CP001779) in the EzBioCloud 16S database (http://www.ezbiocloud.net/eztaxon). We performed PCR and sequencing of housekeeping genes (*groEL* and *gyrB*) using *Streptobacillus* species–specific primers (*5*). BLAST search (http://blast.ncbi.nlm.nih.gov/Blast.cgi) revealed that the *groEL* (GenBank accession no. LC371754) and *gyrB* (GenBank accession no. LC371753) sequences showed 100% identity to the gene sequence of *S. notomytis* KWG2 (522/522 bp and 758/758 bp, respectively) and 99.6% 5(20/522 bp) and 99.9% (757/758 bp) identity, respectively, to the gene sequence of *S. notomytis* AHL_370–1^T^.

We determined antimicrobial susceptibility pattern by broth microdilution. MIC of penicillin was <0.06 µg/mL; cefazolin, <0.5 µg/mL; ceftriaxone, 0.25 µg/mL; vancomycin, <0.25 µg/mL; clarithromycin, 8 µg/mL; minocycline, <0.12 µg/mL; and levofloxacin, <1 µg/mL.

*S. moniliformis* is known to cause rat-bite fever in humans (*6*). To study the association between exposure to rats and *S. notomytis* infection, we visited the patient’s house after her death and captured 2 rats (*Rattus rattus*), from which we collected stool and intraoral and rectal swab samples. On the same day, we brought the specimens at room temperature to our laboratory and performed bacteriological cultures in 5% sheep blood agar, incubated at 37°C under 5% CO_2_; the specimens did not grow *Streptobacillus*. We performed nested PCR with DNA extracted from each specimen, amplified the 16S rRNA gene using the universal primer pair 27F and 1492R, and performed nested PCR using the amplicons from the first PCR as templates, with the *Streptobacillus*-specific primers sbmF (5′-GAGAGAGCTTTGCATCCT-3′) and sbmR (5′-GTAACTTCAGGTGCAACT-3′) (*7*). Only 1 rat’s intraoral specimen yielded PCR products, and the sequence of the amplicon by nested PCR showed 100% identity (1,089/1,089 bp) to *S. notomytis* AHL_370–1^T^.

Since 2014, a total of 4 novel *Streptobacillus* species have been reported. Whether these new species have recently emerged or existed previously is uncertain. In 2014, Eisenberg et al. identified 2 isolates recovered from rats in 2008 as *S. notomytis* (*2*); it is possible that *S. notomytis* may have been prevalent but underrecognized in Japan because identification is difficult by conventional methods (*2*). Fukushima et al. reported that 16S rRNA sequencing identified an isolate obtained from a rat-bite fever patient as *S. notomytis,* not *S. moniliformis* as originally identified (*8*). By detecting *S. notomytis* from the rats captured in this patient’s house, we support a potential epidemiologic link between rat exposure and human infection.

## References

[1] Woo PC, Wu AK, Tsang CC, Leung KW, Ngan AH, Curreem SO, et al. *Streptobacillus hongkongensis* sp. nov., isolated from patients with quinsy and septic arthritis, and emended descriptions of the genus *Streptobacillus* and *Streptobacillus moniliformis.* Int J Syst Evol Microbiol. 2014;64:3034–9. 10.1099/ijs.0.061242-0 24912824

[2] Eisenberg T, Glaeser SP, Ewers C, Semmler T, Nicklas W, Rau J, et al. Streptobacillus notomytis sp. nov., isolated from a spinifex hopping mouse (Notomys alexis Thomas, 1922), and emended description of Streptobacillus Levaditi et al. 1925, Eisenberg et al. 2015 emend. Int J Syst Evol Microbiol. 2015;65:4823–9. 10.1099/ijsem.0.000654 26438009

[3] Eisenberg T, Glaeser SP, Nicklas W, Mauder N, Contzen M, Aledelbi K, et al. *Streptobacillus felis* sp. nov., isolated from a cat with pneumonia, and emended descriptions of the genus *Streptobacillus* and of *Streptobacillus moniliformis.* Int J Syst Evol Microbiol. 2015;65:2172–8. 10.1099/ijs.0.000238 25858245

[4] Eisenberg T, Imaoka K, Kimura M, Glaeser SP, Ewers C, Semmler T, et al. *Streptobacillus ratti* sp. nov., isolated from a black rat (*Rattus rattus*). Int J Syst Evol Microbiol. 2016;66:1620–6. 10.1099/ijsem.0.000869 26705259

[5] Eisenberg T, Ewers C, Rau J, Akimkin V, Nicklas W. Approved and novel strategies in diagnostics of rat bite fever and other *Streptobacillus* infections in humans and animals. Virulence. 2016;7:630–48. 10.1080/21505594.2016.1177694 27088660 PMC4991327

[6] Eisenberg T, Nicklas W, Mauder N, Rau J, Contzen M, Semmler T, et al. Phenotypic and genotypic characteristics of members of the genus *Streptobacillus.* PLoS One. 2015;10:e0134312. 10.1371/journal.pone.0134312 26252790 PMC4529157

[7] Elliott SP. Rat bite fever and *Streptobacillus moniliformis.* Clin Microbiol Rev. 2007;20:13–22. 10.1128/CMR.00016-06 17223620 PMC1797630

[8] Fukushima K, Yanagisawa N, Imaoka K, Kimura M, Imamura A. Rat-bite fever due to *Streptobacillus notomytis* isolated from a human specimen. [Epub 2017 Nov 27]. J Infect Chemother. 2018;24:302–4. 10.1016/j.jiac.2017.10.018 29191371

